# Next-generation sequencing from bulked segregant analysis identifies a dwarfism gene in watermelon

**DOI:** 10.1038/s41598-018-21293-1

**Published:** 2018-02-13

**Authors:** Wei Dong, Defeng Wu, Guoshen Li, Dewei Wu, Zicheng Wang

**Affiliations:** 1 School of Life Science, Henan University, Plant Genetics Laboratory, Kaifeng, Henan 475004 People’s Republic of China; 2 School of Physical Education, Henan University, Kaifeng, Henan 475001 People’s Republic of China; 3 Jiangsu Provincial Key Laboratory of Crop Genetics and Physiology, Yangzhou University, Yangzhou, Jiangsu 225009 People’s Republic of China

## Abstract

Dwarfism is one of the most valuable traits in watermelon breeding mainly because of its contribution to yield as well as the decreased labor required to cultivate and harvest smaller plants. However, the underlying genetic mechanism is unknown. In this study, a candidate dwarfism gene was identified by applying next-generation sequencing technology to analyze watermelon plants. We completed a whole-genome re-sequencing of two DNA bulks (dwarf pool and vine pool) generated from plants in an F_2_ population. A genome-wide analysis of single nucleotide polymorphisms resulted in the detection of a genomic region harboring the candidate dwarfism gene *Cla010726*. The encoded protein was predicted to be a gibberellin 20-oxidase-like protein, which is a well-known “green revolution” protein in other crops. A quantitative real-time PCR investigation revealed that the *Cla010726* expression level was significantly lower in the dwarf plants than in the normal-sized plants. The SNP analysis resulted in two SNP locating in the *Cla010726* gene promoter of *dsh* F_2_ individuals. The results presented herein provide preliminary evidence that *Cla010726* is a possible dwarfism gene.

## Introduction

Watermelon (*Citrullus lanatus*) is among the top five most consumed fresh fruits worldwide, accounting for 7% of the global area devoted to vegetable production^1^. Dwarfism is one of the most valuable traits in watermelon breeding because of its positive effect on yield as well as the associated decreased labor required for cultivating and harvesting the crop. In watermelon, two allelic genes, *dw-1* and *dw-1*^*s*^, and two independent loci, *dw-2* and *dw-3*, have been reported to confer dwarfism^2–5^. Additionally, the cucumber (*Cucumis sativus* L.) genes *cp*, *cp-2*, and *scp* have been identified as responsible for dwarfism-related plant architecture^6–9^. Meanwhile, in tropical pumpkin (*Cucurbita moschata* Duch.) and squash (*Cucurbita pepo* L.), the dwarfism of the vines is regulated by the *Bu* gene^10–13^. Although many studies have been conducted on dwarfism traits in cucurbitaceae plants, the responsible genes have not been cloned^9,14,15^.

Dwarf plant mutations have been important for elucidating the regulatory molecular mechanisms underlying plant growth and development^16^. The main causes of dwarfism have been mutations in hormone biosynthesis or signal transduction pathway-related genes affecting the production of gibberellin (GA)^17–19^, cytokinin^20^, brassinosteroids^21,22^, and other key hormones influencing plant growth and development. Additionally, abnormally developed plant cell membranes or walls can also lead to dwarfism in plants^23,24^.

With the release of sequenced genomes, the combined application of bulked segregant analysis (BSA) and next-generation sequencing technology represents a new way to accelerate the identification of candidate genes controlling important agronomic traits in various crops^25–27^. In 2013, a high-quality draft genome sequence of the Asian watermelon cultivar ‘97103’ (2*n = *2 ×  = 22) was produced. The draft sequence included 23,440 predicted protein-coding genes^1,28,29^, and represented an important resource for plant researchers, particularly those interested in the genetic improvement of crops. The objective of this study was to identify the dwarfism gene in the *dsh* mutant watermelon line, which was derived from line ‘I911’ (Code I911; inbred hybrid selected in the seventh generation). Compared with the ‘I911’ plants, the *dsh* plants had short vines and stems, numerous branches and flowers, and small fruits. The ratio of long-vine to short-vine plants for the F_2_ and BC_1_ populations conformed to Mendel’s segregation ratios of 3:1 and 1:1, respectively^30^. Thus, we concluded that the dwarfism trait is a qualitative characteristic (QC) controlled by a single gene. We re-sequenced the whole genome of two DNA bulks (i.e., dwarf pool and vine pool) developed from plants in an F_2_ population. A genome-wide analysis of single nucleotide polymorphisms (SNPs) enabled the detection of a genomic region harboring the dwarfism gene. Results from this study provide preliminary evidence that *Cla010726* is a possible candidate gene encoding the dwarfism trait.

## Methods

### Plant materials and phenotyping for dwarfism

Two watermelon inbred lines, *dsh* and ‘I911’, were used as the parents to generate the F_1_ and F_2_ populations. The *dsh* watermelon plant (female parent) is a bush with a short vine, short internodes, thin stems, numerous branches, and small leaves, flowers, and fruits. The 139 F_2_ individuals and 20 parent plants were grown and evaluated at the Henan University Genetics and Breeding Base in the spring of 2016.

### Data generation

Genomic DNA was extracted from fresh leaves collected from the 2 parent plants as well as the 30 dwarf and 30 vine plants using the CTAB method^31^ for a subsequent QC-sequencing (QC-seq) analysis. The degradation and contamination of the extracted DNA were monitored on 1% agarose gels, while the DNA purity was checked using the NanoPhotometer® spectrophotometer (IMPLEN, CA, USA). The DNA concentration was measured using the Qubit® DNA Assay Kit and the Qubit® 2.0 Fluorometer (Life Technologies, CA, USA). For the QC-seq analysis, two DNA pools, namely the dwarf pool (D-pool) and vine pool (V-pool), were constructed by mixing an equal amount of DNA from the 30 dwarf and 30 vine F_2_ plants collected in the autumn of 2016. A total of 1.5 μg DNA per sample was used as the input material.

Sequencing libraries were generated using the TruSeq Nano DNA HT Sample Preparation Kit (Illumina, USA) following the manufacturer’s recommendations. Separate index codes were added to attribute sequences to different samples. Briefly, DNA samples were sonicated to generate 350-bp fragments, which were then end-repaired, A-tailed, and ligated with the full-length adapter for Illumina sequencing by PCR amplification. Finally, the PCR products were purified using the AMPure XP system and the size distribution of the libraries was analyzed with the Agilent 2100 Bioanalyzer. The libraries were quantified by quantitative real-time (qRT)-PCR and then sequenced using the Illumina HiSeq 4000 platform to generate 150-bp paired-end reads, with an insert size around 350 bp.

### Data analysis

To ensure reads were reliable and to prevent any artificial bias in the following analyses, the raw reads underwent a series of quality control procedures using in-house C scripts. Raw reads were removed based on the following criteria: (1) reads with ≥10% unidentified nucleotides; (2) reads with >50% bases having a phred quality score <5; (3) reads with >10 nucleotides aligned to the adapter, allowing ≤10% mismatches; (4) putative duplicates generated by the PCR amplification during the library construction process. The BWA program was used to align the D-pool and V-pool clean reads against the reference genome sequence^32^. Alignment files were converted to BAM files using the SAMtools program^33^. Additionally, potential PCR duplications were removed using the SAMtools command “rmdup”. If multiple read pairs had identical external coordinates, only the pair with the highest mapping quality was retained.

### Analyses of quality traits with SNP and InDel markers

All samples underwent variant calling using the Unified Genotyper function of the GATK program^34^. The SNPs and InDels were filtered using the Variant Filtration parameter of GATK. ANNOVAR, which is an efficient software tool, was used to annotate the SNPs or InDels based on the GFF3 files for the reference genome^35^. The homozygous SNPs/InDels between two parents were extracted from the vcf files. The read depth information for the homozygous SNPs/InDels in the D-pool and V-pool was obtained to calculate the SNP/InDel index^25^. We used the dwarf genotype of the parent as the reference and for analyzing the read number for the D-pool and V-pool. We then calculated the ratio of the number of different reads to the total number of reads, which represented the SNP/InDel index of the base sites. We filtered out those points in which the SNP/InDel index in both pools was <0.3. Sliding window methods were used to determine the SNP/InDel index of the whole genome. The average of all SNP/InDel indices in each window was used as the SNP/InDel index for that window. We usually used a window size of 1 Mb and a step size of 10 kb as the default settings. The difference between the SNP/InDel index of two pools was calculated as ΔSNP/InDel index.

### Expression analysis of candidate dwarfism genes by quantitative real-time PCR

We investigated the expression patterns of *Cla010721*, *Cla010725*, *Cla010726*, and *Cla010750* using qRT-PCR. The *dsh* and ‘I911’ tissue culture seedlings were grown for 10 and 30 days. Each collected sample represented one replicate. Total RNA was extracted from all samples using the Trizol reagent (Invitrogen, Carlsbad, CA, USA). The PrimeScript™ RT reagent Kit with gDNA Eraser (TaKaRa, Dalian, China) was used to reverse transcribe cDNA from the extracted total RNA. The resulting cDNA samples were analyzed by qRT-PCR in a 20-μl reaction volume containing 10 μl SYBR Premix Ex Taq II (TaKaRa). The *ClYLS8* gene (encoding yellow-leaf-specific protein 8) was included as a control for normalizing gene expression data (Kong *et al*., 2014). The qRT-PCR was completed using an ABI 7500 Fast Real-time PCR system. There were five biological repeats for dsh and ‘I911’. Each sample was analyzed three times (i.e., technical replicates). The primers used to detect the transcripts of structural and regulatory genes are listed in Table 1.

**Table 1:** Sequences of the quantitative real-time PCR primers

Primer name	Forward primer sequence	Reverse primer sequence
Cla010721	GAGCAACTGGGGATGGCGACAT	GGCAAGCACCGGCATGAGTA
Cla010725	GGCCGCCAACGTCTACATGCTT	CGCCAATTCCAACGCAGAGT
Cla010726	CGACTTAGGGTTTACGGAAC	GCTCTCAAAATTATCTCCCA
Cla010750	CATACTCATCCTTTATCACC	TATATGTTGCAGATCGCTTT
ClYLS8	AGAACGGCTTGTGGTCATTC	GAGGCCAACACTTCATCCAT

### Analysis of the SNP in the promoter of the candidate dwarfism gene

Genomic DNA was extracted from fresh leaves collected from the 30 dwarf and 30 vine plants using the CTAB method^31^. The degradation and contamination of the extracted DNA were monitored on 1% agarose gels, while the DNA purity was checked using the NanoPhotometer® spectrophotometer (IMPLEN, CA, USA). The fragments with SNP were amplified by PCR using the following primer pair: forward, 5′-TGTTGAAATTTGGTGACGAGGT -3′, and reverse, 5′- TGAATTAAACGTTTCGGGCAC -3′ in a 20-μl reaction volume containing 0.2 μl Ex Taq (TaKaRa). And then the PCR products were sequenced.

## Results

### Morphology of dwarf watermelon plant

**Figure 1:**
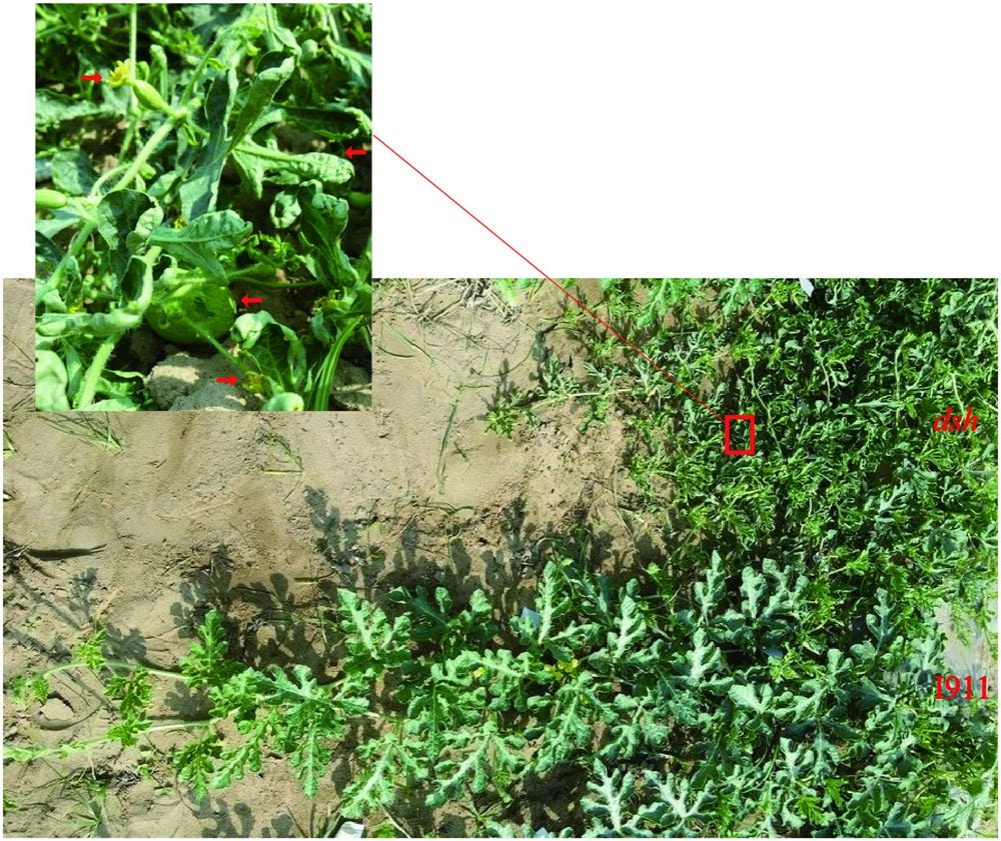
Comparison of the morphological indices between the ‘dsh’ mutant and the wild-type ‘I911’.

After years of screening and cultivating, the *dsh* dwarf watermelon plant was detected in an inbred watermelon line derived from ‘I911’ in July 2009. There were considerable morphological differences between *dsh* and ‘I911’ plants (Fig. 1). The *dsh* plants produced short vines and stems, many branches and flowers, and small fruit, which was unlike the ‘I911’ plants. Moreover, the leaf edges of *dsh* plants were curled. The results showed that F1 generation all developed long vines, whereas the ratio of long-vine to short-vine plants for the F_2_ populations conformed to Mendel’s segregation ratios of 3:1 (maximum *χ*^2^ value as 0.54, *P* > 0.05).

### QC-seq identified four candidate genes controlling dwarfism on chromosome 7

**Figure 2:**
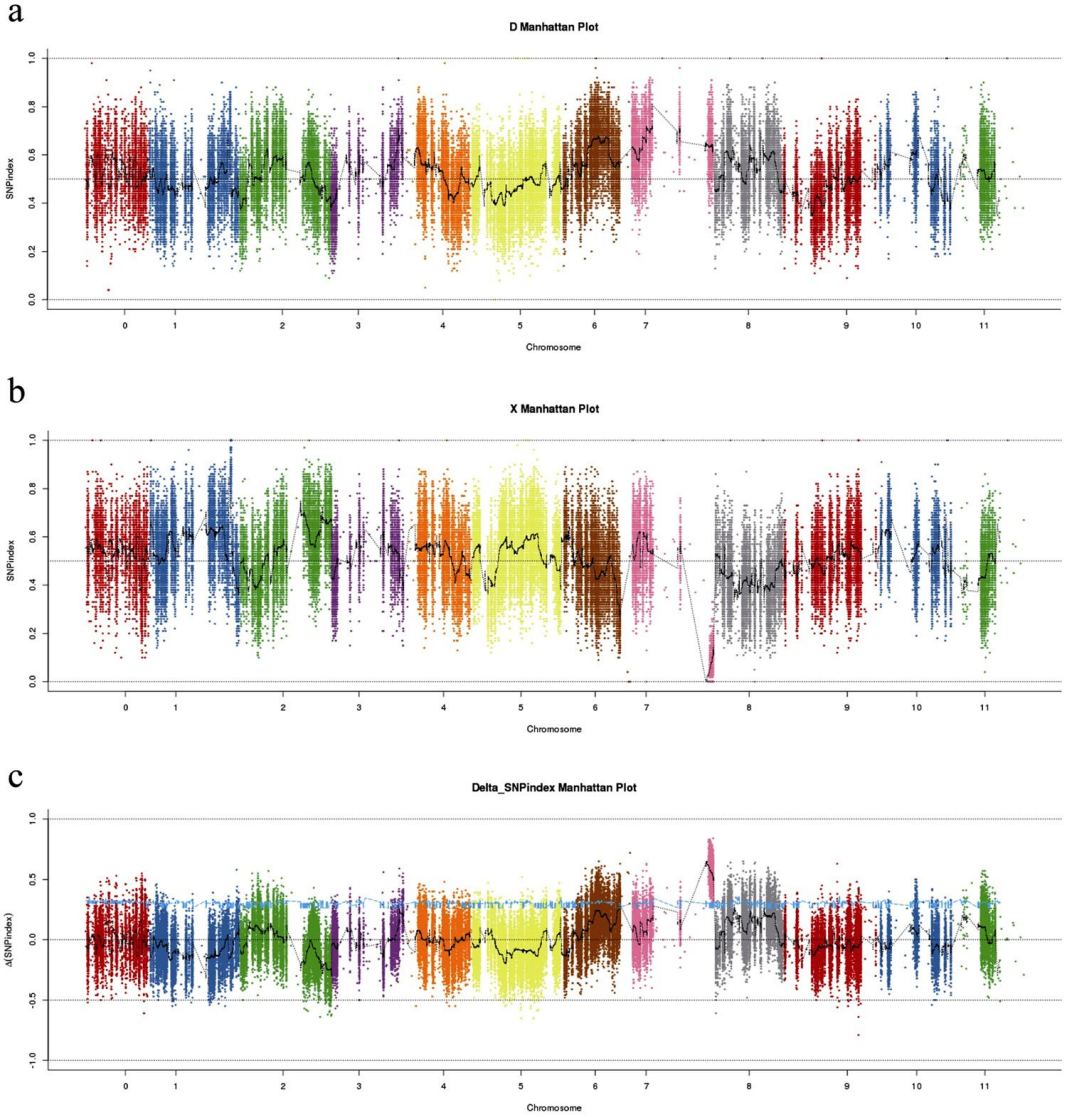
SNP-index graphs of the D-pool (**a**), V-pool (**b**), and Δ (SNPindex) (**c**) for the QC-seq analysis. The x-axis represents the position of seven chromosomes and the y-axis represents the SNP-index, which was calculated based on a 1 Mb window with a 10 kb step.

Illumina high-throughput sequencing resulted in 18,371,510,400 bp and 15,618,092,700 bp reads for the D-pool (45.94 × average depth coverage or 99.68% coverage) and V-pool (39.62 × average depth coverage or 99.65% coverage), respectively. These reads were aligned to the 355,247,419 bp reference watermelon genome and 352,235 SNPs were identified which included homozygous SNP and heterozygous SNP^1^. We used the dwarf genotype of the parent as the reference to calculate the homozygous SNP index of 97,539 polymorphic markers between the two offspring. After screening, 97,186 polymorphic markers were obtained after filtration (Supplementary Table S1). An average SNP-index was computed in a 1 Mb window using a 10 kb step. The SNP-index graphs were generated for the D-pool (Fig. 2a) and V-pool (Fig. 2b) by plotting the average SNP-index against the position of each step in the genome assembly. By combining the information for the SNP-index in the D-pool and V-pool, the Δ (SNP-index) was calculated and plotted against the genome positions (Fig. 2c).

The Δ (SNP-index) value should be significantly different from 0 if a genomic region harbors a target gene. At the 95% significance level, 41 polymorphic marker loci were selected (Supplementary Table S2). At the 99% significance level, only one genomic region on chromosome 7 (27.66–30.61 Mb) had a Δ (SNP-index) value that was significantly different from 0 (Supplementary Table S3). The results of ANNOVAR’s annotation indicated that there were four candidate watermelon genes responsible for the dwarfism phenotype in the 27.66–30.61 Mb region on chromosome 7. The four candidate watermelon genes were *Cla010721*, *Cla010725*, *Cla010726* and *Cla010750*. The SNP of *Cla010721* located in *exon* which was nonsynonymous mutation. And the others located in the promoter (Supplementary Table S4). But they all did not change the sequence of amino acids. It was predicted that Cla010721 was an asparaginase-like protein, Cla010725 was a sugar transporter, Cla010726 was a GA20-oxidase-like protein and Cla010750 was a FAR1-related protein.

### Identification of the dwarfism gene

**Figure 3:**
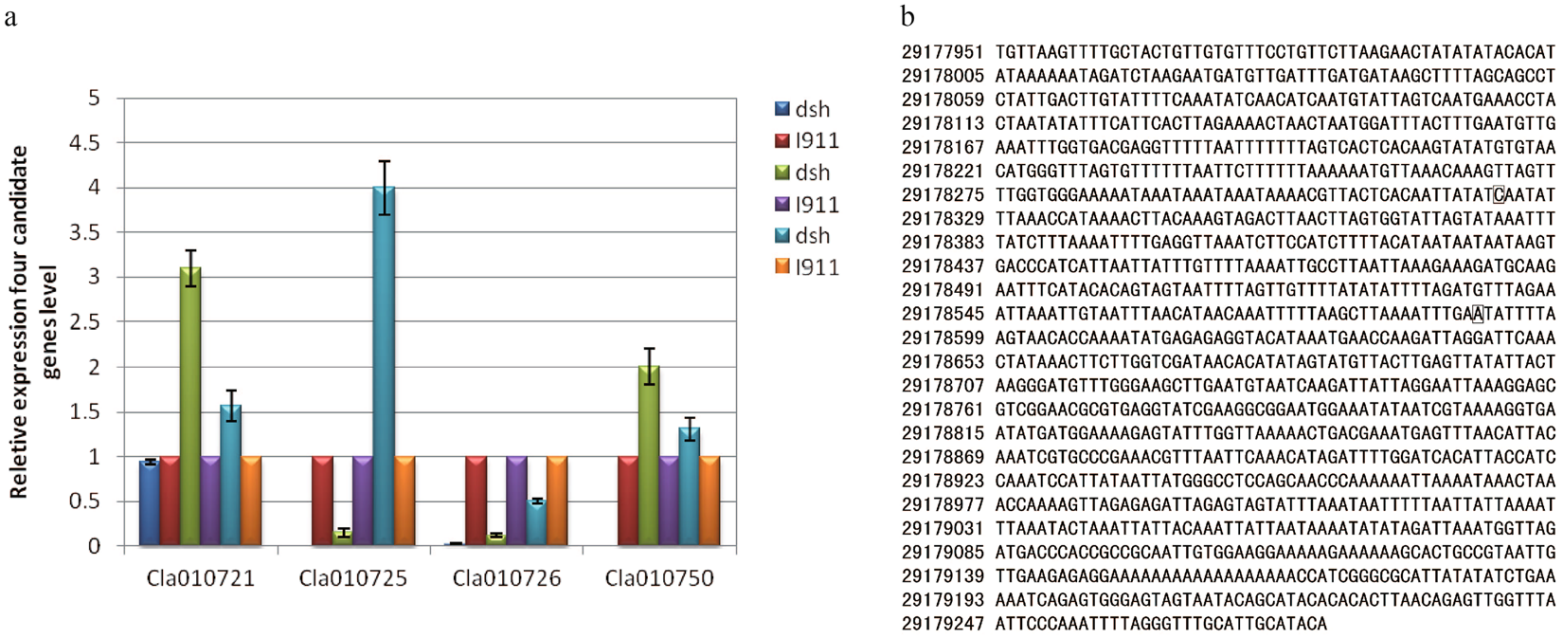
Expression of watermelon four candidate genes and nucleotide sequence of *Cla010726* gene promoter. (**a**) Relative expression levels of four candidate genes in *dsh* and ‘I911’ plants. Data are presented as the mean of three independent measurements. Error bars represent the standard deviation of the mean values. (**b**) Nucleotide sequence of the 1329 bp upstream region of *Cla010726*. The SNP site was in bold.

We predicted the presence of four candidate dwarfism genes in a 27,800-kb region of watermelon chromosome 7. The highest Δ (SNP-index) value existed in the 1328 bp upstream region of the *Cla010726* gene (Fig. 3a, Supplementary Table S3). The *Cla010726* gene also appeared promising based on the gene annotation result. Cla010726 was predicted to be a GA20-oxidase-like protein that contains InterPro domain IPR005123 (oxoglutarate and iron-dependent oxygenase). The results of a BLAST alignment revealed that the identity between the *Cla010726* and the *C*. *sativus GA20-oxidase 2-like* gene was as high as 86%. Additionally, the sequence identity of the encoded proteins was 82.55%. Moreover, the *Arabidopsis thaliana* GA20ox family includes GA20-oxidase 1 and GA20-oxidase 2. The results of a protein BLAST alignment indicated that the sequence identity between Cla010726 and AtGA20ox1 was 30.50% and between Cla010726 and AtGA20ox2 was 29.60%. An important function of GA20ox in many plant species involves regulating GA concentrations. Thus, we proposed that *Cla010726* is a *GA20ox* homolog in watermelon and named this gene *ClaGA20ox* (*C*. *lanatus gibberellin 20-oxidase*). This gene represents the most likely candidate gene responsible for the dwarfism of watermelon plants.

We examined the four candidate watermelon genes *Cla010721*, *Cla010725*, *Cla010726* and *Cla010750* expression patterns in the two parental lines by qRT-PCR to assess whether the genes expression level influences the development of the dwarfism phenotype (Fig. 3a). The *Cla010726* expression level was considerably higher in ‘I911’ plants than in *dsh* plants (*P < *0.05), further suggesting that *ClaGA20ox* may be responsible for the dwarfism in watermelon plants. We examined the SNP of *Cla010726* gene promoter in the F_2_ population by sequencing. The SNP analysis resulted in two SNP locating in the *Cla010726* gene promoter of *dsh* F_2_ individuals (Fig. 3b).

## Discussion

Dwarfism is an important trait in watermelon breeding. In this study, *dsh* was a dwarf mutant that had been identified in an inbred watermelon line derived from ‘I911’. Advantages of this dwarf mutant include its high growth efficiency, low soil fertility requirements, and the fact it can be grown at a high planting density. This relatively new germplasm resource should be further developed in the future. However, the genetic mechanism underlying the dwarfism phenotype remains unknown. We completed a QC-seq analysis to characterize the dwarfism in watermelon plants using an F_2_ mapping population. This method combined a high-throughput whole-genome re-sequencing with a bulked-segregant analysis, which represents a quicker and more efficient method of identifying a target gene. Functional orthologs of this gene with mutations have been selected as so-called “green revolution genes” in rice and barley.

Our analyses identified *Cla010726* on watermelon chromosome 7 as a potential gene responsible for the observed dwarfism. This gene is a homolog of *GA20ox* genes found in many plant species. Thus, we designated this gene *ClaGA20ox*. A previous study revealed that GA20ox is a key oxidase enzyme that contributes to GA biosynthesis by catalyzing the conversion of GA12 and GA53 to GA9 and GA20, respectively, *via* a three-step oxidation at C-20 of the GA skeleton^19^. Five copies of *GA20ox* genes have been detected in *A*. *thaliana*^36^. Mutations to this gene have different effects on overall plant growth. Specifically, the *ga20ox1* line exhibits a semi-dwarf phenotype, whereas *ga20ox2* plants are only slightly smaller than wild-type plants^19^. Additionally, *AtGA20ox1* is an ortholog of the rice *SD1* (semi-dwarf l) gene and barley *sdw1*/*denso* green revolution genes^37^. Four *GA20ox-like* genes have been identified in the rice genome^18^. *OsGA20ox2* (or *SD1*) is a well-known gene that has been studied in green revolution rice varieties^38,39^. It is one of the most important genes deployed in modern rice breeding programs^9,40^. The *sdw1*/*denso* gene has been one of the most successful semi-dwarfing genes used in barley breeding worldwide^41–43^. Furthermore, one of the *HvGA20ox2* genes was identified as a candidate gene for *sdw1*/*denso*, which is an ortholog of the rice *sd1* gene^44,45^. The first *GA20ox* gene was isolated from pumpkin (*Cucurbita maxima* L.)^46^. The *GA20ox* gene associated with a dwarf vine was also anchored in pumpkin (*C*. *maxima* D.)^15^ according to a high-density genetic map. Among the known GA20-oxidases, only that from developing pumpkin seeds has been shown to produce biologically inactive GA as the major product^47^. Transgenic lettuce carrying the pumpkin *GA20ox* exhibited a dwarfism phenotype in the T_2_ generation plants^48^. Therefore, it is reasonable to postulate that *ClaGA20ox* is a viable candidate gene responsible for dwarfism in watermelon plants. However, further evidence is needed to functionally validate this. Accordingly, we are currently generating *ClaGA20ox* overexpressing and knock-out mutant lines for a subsequent examination of gene expression and function. Characterizing the mechanism underlying the dwarfism of *dsh* plants will likely be relevant for future molecular breeding efforts.

## Electronic supplementary material

Dataset 1

Dataset 2

Dataset 3

Dataset 4
